# The Impact Behaviour of Crab Carapaces in Relation to Morphology

**DOI:** 10.3390/ma13183994

**Published:** 2020-09-09

**Authors:** Puspa Restu Sayekti, Fahrunnida, Gabrielis Cerniauskas, Colin Robert, Bambang Retnoaji, Parvez Alam

**Affiliations:** 1 School of Engineering, Institute for Materials and Processes, The University of Edinburgh, Edinburgh EH8 9YL, UK; puspa.restu@gmail.com (P.R.S.); fnida.biougm@gmail.com (F.); g.cerniauskas@ed.ac.uk (G.C.); Colin.Robert@ed.ac.uk (C.R.); 2 Laboratory of Animal Structure and Development, Department of Tropical Biology, Faculty of Biology, Universitas Gadjah Mada, Yogyakarta 55281, Indonesia; bambang.retnoaji@ugm.ac.id

**Keywords:** crab carapace, mechanical properties, mechanical behaviour, impact resistance, biomimetic design, comparative biomechanics, topology

## Abstract

Brachyuran crab carapaces are protective, impact-resistant exoskeletons with elaborate material microstructures. Though several research efforts have been made to characterise the physical, material and mechanical properties of the crab carapace, there are no studies detailing how crab morphologies might influence impact resistance. The purpose of this paper is to characterise and compare Brachyuran crab carapace morphologies in relation to their impact properties, using opto-digital, experimental and numerical methods. We find that crab carapaces with both extended carapace arc-lengths and deep carapace grooves lose stiffness rapidly under cyclic impact loading, and fail in a brittle manner. Contrarily, carapaces with smaller arc lengths and shallower, more broadly distributed carapace grooves are more effective in dissipating stresses caused by impact throughout the carapace structure. This allows them to retain stiffness for longer, and influences their failure mode, which is ductile (denting), rather than brittle fracture. The findings in this paper provide new bioinspired approaches for the geometrical designs by which means material failure under cyclic impact can be controlled and manipulated.

## 1. Introduction

Comprising approximately 7000 identified species within 98 families, Brachyura (order Decapoda, class Malacostraca) are among the most diverse crustacean infraorder, inhabiting marine, freshwater, and terrestrial ecosystems [1]. Brachyurans (true crabs) are the most successfully evolved crustacean [2], possessing highly organised physiological and morphological characteristics including limb regeneration, a complex carapace (shell) microstructure, and effective locomotion [2]. Brachyuran crabs live abundantly in different habitats along coastlines and littorals, and their carapace structures are eco-physiologically adapted for defence to predatory attack [3,4]. One common carapace adaptation is a helicoidal (Bouligand) architecture comprised of mineralised chitin-protein fibres [5,6,7]. A through-thickness Bouligand arrangement optimises impact energy dissipation by increasing the effective travel path of a propagating crack [8]. This coupled to extra forces required to cause high aspect ratio calcite crystals to slip, maximises the final fracture energy required to break through the crab shell [9,10]. The strength and toughness of crab shells are affected by hydration, which correlates to hydrogen bonding within the material. The wet shells of sheep crabs (*Loxorhynchus grandis*) [11] and Shanghai hairy (Chinese mitten) crabs (*Eriocheir sinensis*) for example, are significantly stronger and tougher than dry shells. Hydrogen bonding heightens under aqueous conditions and this in turn strengthens the bonding between individual components within the mineralised chitin-protein fibres. Wet environments are therefore mechanically favourable for crab shells, optimising their utility as defence tools and increasing their chances of survival when attacked.

Certain morphological features have been identified as important for shell strength. The width of snow crabs (*Chionoecetes opilio*) for example, affects carapace load-bearing capacity in compression, with wider carapaces generally exhibiting load-bearing superiority to narrower carapaces [12]. Age has been identified as another parameter of importance, since crabs moult in order to grow. Moulting entails the loss of the hard protective carapace, which reforms with the crab. Brachyurans typically develop initial soft phases, followed by papery phases before the carapace reaches its final postmoult hardened phase. The hardened carapace phase is superior in flexion to the prior soft and papery phases, which in the blue crab (*Callinectes sapidus*) is reported as being 10^4^ fold higher than the papery phase [13]. In its hardened phase, the nanoscale properties of the carapace are a function of the local structures of the mineral phases, which affects local stiffness and hardness gradients, as evidenced in the edible crab, *Cancer pagarus*, [14].

Crabs regulate their respiration and excretion [15], inter-moult durations [16], and mating behaviours [17], each of which has been identified as an eco-physiological adaptation for defence against predators. Predators often repetitively impact a crab shell, often at its apex, to break through to the edible underlying soft-tissue [18]. As such, certain crab species may possess geometrical adaptations to improve shell penetration resistance during impact. To date, research on the mechanical properties of crab shells has been focussed on variables such as age, moult cycle, localised properties, materials, and microstructure. The protective role of carapace geometry against impact forces during a predatory attack, is not well understood.

In this paper, we research the impact resistance of five Brachyuran crab species in relation to the geometrical characteristics of their carapaces: *Leptodius sanguineus* (H. Milne Edwards, 1834), *Thalamita* sp. (either of *Thalamita danae* (Stimpson, 1858) or *Thalamita prymna* (Herbst, 1803)), *Calappa hepatica* (Linnaeus, 1758), *Pilumnus vespertilio* (Fabricius, 1793), and *Uca tetragonon* (Herbst, 1790). Both *Thalamita* sp. of crab are similarly sized, live in similar environments and have very similar carapace structures. Since our work is focussed on carapace morphology, we researched both species listed within the genera.

*L. sanguineus* (rock crabs) inhabit microhabitats, examples of which include (under or inside) pieces of coral rubble, rock crevices, and algal assemblages [19,20]. Rock crabs are also exposed or sheltered on the rocky shore at low tide along the intertidal reef shoreline [21,22,23,24]. *L. sanguineus* possesses a relatively flat carapace with a fine granular pattern, which they may use for both camouflage and defence. These crabs usually also move more slowly when under predatory attack. *Thalamita* sp. (spiny rock crabs) are ambush predators and scavengers [25] found in shallow water habitats such as mangrove swamps. When submerged under higher tide conditions, *Thalamita* sp. tends to seek shelter in crevices or burrows to avoid swimming predators such as fishes, squids and other Portunid crabs. They are also able to migrate to other areas when tides are sufficiently low; however, this exposes the crabs to predators. When under threat from flying predators such as the crab plover (*Dromas ardeola*), these crabs bury themselves under the mud or sand [26]. *Calappa* sp. (box crab) are commonly found in rocky and coral reefs areas on the sandy or mud-sand flats of the intertidal zone [27]. Box crabs possess sand-like carapaces, which are beneficial for camouflage in sandy surroundings. *Pilumnus* sp. crabs are covered with dense hairs, which are essentially chitin setae typical of crab species from the same Heterotremata [28]. These crabs live in silt and rocky littorals, which allows them to easily occupy holes or crevices when under predatory attack [21]. *Pilumnus* sp. crabs are generally nocturnally active animals. *Uca* sp. (fiddler crabs) are deposit feeders [29] typically found in mudflats where they build tunnels to escape from predators and evade desiccation due to high ambient temperatures [30].

## 2. Materials and Methods

### 2.1. Collection and Identification of Crabs

Five genera of crabs (comprising six species) were collected from Sombu (05°16′12.2” S latitude; 123°31′08.8” E longitude) and Liya (05°23′12.1” S latitude; 123°35′38.4” E longitude), Wangi-Wangi Island, Wakatobi, Southeast Sulawesi, Indonesia between January 2–4, 2017. The crabs purposively sampled along the coastlines. The crab genera were identified by examining the morphological characteristics of their carapaces, chelae, pereopods, and their dorsal/ventral faces, and comparing against taxonomy classification keys from [31] and [32]. Crabs were euthanised using diluted clove oil containing eugenol (0.125 mL/L) in accordance with the American Veterinary Medical Association (AVMA) Guidelines for the Euthanasia of Animals Section S.6.3: Aquatic Invertebrates. Euthanised crabs were then digitally documented and initially fixed in 96% ethanol solution, after which they were stored for further research in 70% ethanol solution.

### 2.2. 3D Imaging and Morphometric Analyses

**Figure 1:**
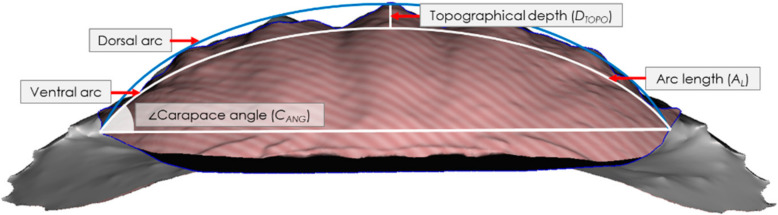
Intersecting arcs and lines used to determine the arc length, *A_L_*, the carapace angle, *C_ANG_*, and the topographical depth, *D_TOPO_* (maximum distance between the dorsal and ventral arcs).

Each crab was scanned prior to testing using an Einscan Pro 2X Shining 3D scanner (Shining 3D Tech. Co., Ltd., Hangzhou, China) with a post-calibration accuracy of 40 µm and the data is gathered using 16 increments (22.5 each) for a full 360° rotation. Point cloud images were then recorded, processed, edited and exported in a stereolithographic (.stl) file format using Shining 3D software. Post-processing of the .stl files was conducted in Autodesk Meshmixer (Autodesk Inc, San Rafael, CA, USA) and morphological parameters were then measured using ImageJ (NIH, Bethesda, MD, USA) across 2D slices of the carapaces to yield morphometric data. We measured the following morphological parameters: carapace thickness, carapace arc length, angle, topographical depth, 2D Wenzel roughness, and the arc length normalised topographical depth. The thickness, *t*, was measured using an electronic micrometer (0–25 mm) with an accuracy to 1 µm. Measurements (at least three per sample) were taken, and these were averaged. The carapace arc length, *A_L_*, was determined by intersecting the closest fitting curve with the ventral face of the carapace as observed in 2D from an anterior view sliced through the apex of the carapace, Figure 1, and therewith calculating its length. The carapace angle, *C_ANG_*, was then calculated by intersecting a horizontal linear line through the arc at the ventral-most extents of the carapace/arc intersection and using the angle measurement tool in ImageJ, cf. Figure 1. To calculate the topographical depth, *D_TOPO_*, a second arc was drawn that followed the outermost dorsal curvature from the apex peaks of the carapace to the original arc at its ventral-most extents at the carapace/arc intersection. The maximum distance between the two intersecting arcs provided the value for the topographical depth, cf. Figure 1. The 2D Wenzel roughness, *W_r_*, was calculated as a ratio between the actual length of the carapace surface and *A_L_*.

### 2.3. Impact Testing

**Figure 2:**
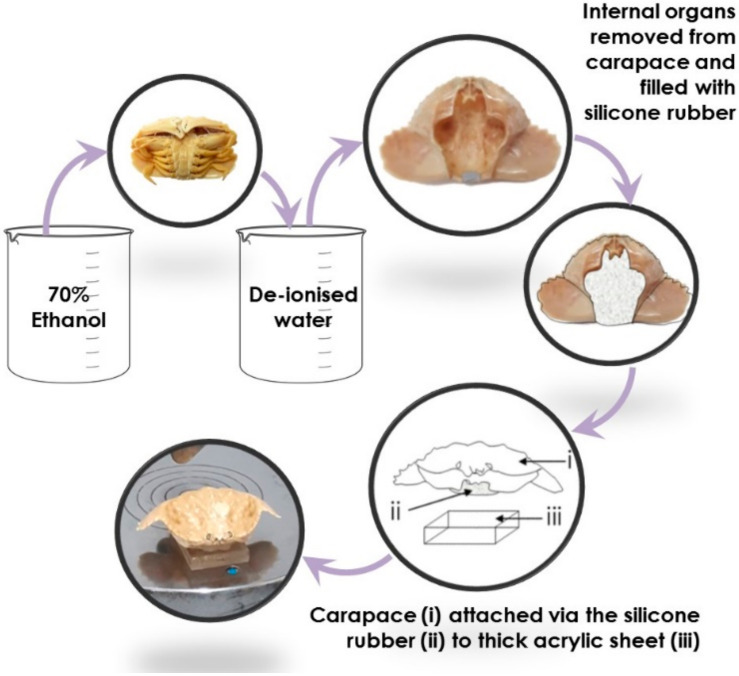
Preparation process of crab carapaces prior to mechanical testing.

Crabs were transferred from 70% ethanol solution to deionised water, where they were soaked for three days. Following their saturation in deionised water, individual carapaces from each crab of each species were separated from the rest of the body by dissection. The carapaces were filled with silicone gel, attached to individual 9 mm thick acrylic blocks (one per carapace), and dried for 24 h, Figure 2. The silicone gel acted as a soft matter control beneath the carapace, thus ensuring that the carapace itself was the focal point during testing, and negating any additional independent variables that may arise through differences in the soft tissues between each genus. The carapace apex, which is known to be a target for repetitive predatorial impact events [18], was marked on each specimen to indicate the precise location for impact testing. Prior to impact testing, the carapace flexural stiffness, *k*, was measured using an Instron 3369 (Instron, Norwood, MA, USA) using a 5 kN load cell. Carapaces attached to 9 mm thick acrylic blocks were flexed at their marked apex point using a three-point bending steel roller.

Drop impact testing was then performed on the wet crab carapaces to determine the impact resistance of each carapace in each genus by measuring the residual stiffness after each impact event. Drop impact testing was conducted using stainless steel balls dropped vertically onto the marked carapace apex. To ensure accurate targeting of the carapace apex, the balls were dropped through appropriately sized glass tubes at initial heights of 132 cm using a 0.132 g steel ball. The post-impact *k* value was measured after every third impact event to determine the residual stiffness of the carapace. If after 500 impact events there was no change in the stiffness of the carapace, testing was continued using 1.043 g steel balls dropped from heights of 151 cm.

### 2.4. Impact Simulations

**Figure 3:**
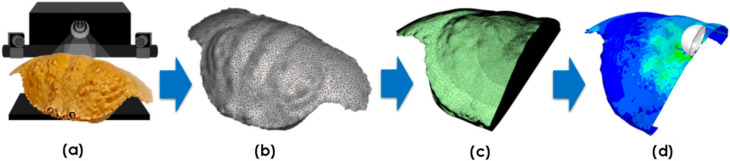
3D scan to FE analysis process: (**a**) point cloud data is obtained and processed; (**b**) surface mesh is edited; (**c**) orphan mesh suitable for FE analysis is generated; (**d**) FE model is defined.

Representative crab shells from each species (excluding *T. prymna*) were used to conduct finite element (FE) simulations. A median (size) crab was chosen from each sample set as the representative. Surface mesh .stl files from the 3D scans (Section 2.1) were post-processed using Autodesk MeshMixer (Autodesk Inc., San Rafael, CA, USA) to create a set of orphan meshes suitable for FE analysis. Mesh density was adjusted and areas of interest (e.g., in the vicinity of the impact point) were re-meshed to increase the accuracy of the simulation. Since the 3D scan data showed the specimens were highly symmetrical, the digitalised models were halved to minimise the computations required. For each simulation, an orphan mesh was imported into Abaqus/CAE (Dassault Systèmes) modelling software. The crab carapace geometry was then defined by assigning shell element properties to the imported mesh. The shell thickness values were matched against the experimental morphometric data. The complete 3D scan to FE simulation process is summarised in Figure 3.

Two parts were considered for discretisation: (1) the projectile (i.e., the steel ball impactor with a diameter of 6.34 mm) and (2) the imported orphan mesh (i.e., the mesh created from the 3D scan of the crab shell). We assume negligible deformation of the steel projectile, which was assigned a shell geometry in Abaqus/CAE (Dassault Systèmes, Vélizy-Villacoublay, France) and discretised with a native mesh made of four-noded quad-dominated (R3D4) elements. The mesh density was chosen to be high enough to capture the ball shape of the impactor, Table 1. This approach eliminates the need for element-level calculations on the projectile geometry, decreasing the overall computational time.

**Figure 4:**
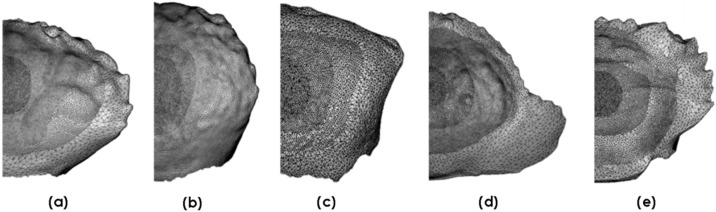
Orphan meshes for each of the five crab species: (**a**) *L. sanguineus*; (**b**) *P. vespertilio*; (**c**) *U. tetragonon*; (**d**) *C. hepatica*; (**e**) *T. danae*.

Figure 4 shows imported meshes for each of the crab genera. These were tailored to enable analyses of features of interest in each of the crab genera. Each mesh was discretised with a finer mesh near the point of impact, and the mesh density gradually decreased concentrically from the impact point towards the edges of the carapace geometry. The three-noded triangular (S3R) shell elements with a reduced integration feature were assigned to the carapaces. The finite strain formulation was chosen since impaction alters the geometry significantly.

An experimentally representative vertical freefall impact simulation was run, comprising a steel ball of 1.043 g accelerating under a gravitational pull from a height of 151 cm. The projectile velocity at impact of 5.44 m/s was calculated based on the kinetic impact energy of the steel ball (15.45 mJ). The mass distribution on the projectile was defined with a point mass at the centre of the projectile and only the movement in the vertical direction (z-axis) was allowed, mimicking thus the kinematics of the steel ball as guided by the glass tube. As such, the impacting steel ball was kinematically limited to a single degree of freedom impacting the dorsal carapace apex.

**Figure 5:**
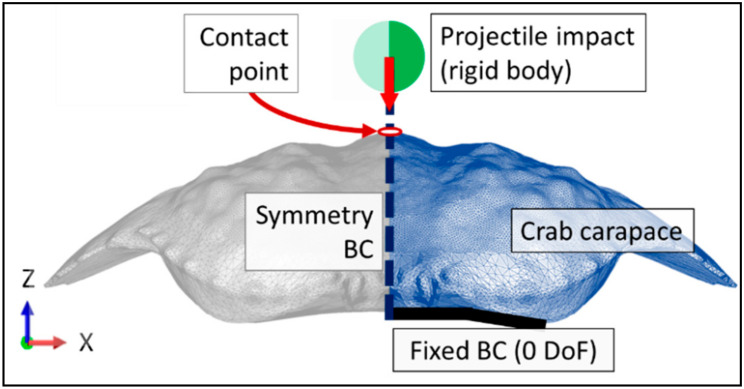
Carapace impact and boundary conditions (denoted by BC) schematic.

The ventral edges of the carapace were fixed with zero degrees of translational and rotational freedom, Figure 5. This was considered to be the most accurate representation of the experimental testing conditions, as the experimental carapaces were affixed to an acrylic plate against their ventral edges, which were in turn the locations for the reaction forces to impact loading. A symmetry boundary condition was applied to the plane of symmetry of both carapace geometries and the projectile geometry, Figure 5.

A general contact algorithm within Abaqus/Explicit was used to describe the surface-to-surface contact interaction between the steel ball and the carapace. A standard Coulomb friction model was used to characterise contact, and a friction coefficient (*μ*) of 0.5 was applied between the steel ball and the carapace. The standard Coulomb friction model assumes that no relative motion occurs if the equivalent frictional stress (*τ_eq_*) is less than the critical stress (*τ_crit_*). The equivalent shear stress term was calculated by combining the two shear stress components (*τ_1_* and *τ_2_*) at the interface, as shown in Equation (1).

(1) $$\tau_{eq}=\sqrt{\tau_{1}^{2}+\tau_{2}^{2}}$$

Meanwhile, the critical stress term (*τ_crit_*) is defined as a product of the contact pressure (*p_c_*) and the friction coefficient (*μ*) (cf. Equation (2)). The model therefore relates the shear stress and the pressure at the point of contact, and determines a transition point between the two surfaces slipping and sticking together.

(2) $$\tau_{crit}=\mu p_{c}$$

Compressive mechanical properties for crab carapaces as reported by Chen et al. [11] were input to the simulation. The carapace is assumed to have identical mechanical properties in the in-plane and out-of-plane directions. An elasto-plastic fracture mechanics (EPFM) model was constructed to simulate brittle failure using the following inputs: Young’s modulus = 1.069 GPa, Poisson’s ratio = 0.3, density = 1.5 g/cc, compressive strength = 57 MPa, shear strength = 9.9 MPa, fracture strain = 0.052. The model yield strength, *σ_y_*, is at 52 MPa, after which it strains plastically, *ε_pl_*, to its compressive strength of 57 MPa as $\sigma_{pl}=23.434\varepsilon_{pl}^{0.6117}$, where *σ_pl_* is the stress during plastic straining (following the experimental work of Chen et al. [11]).

Damage caused by the impacting body on the target body is described by element deletion, which captures the combined effect of both ductile and shear damage. The overall damage variable was used to assess stiffness degradation in an element at the location of damage, and this occurred when a shell element is fully damaged and the initial stiffness is lost. This approach was used to ensure convergence beyond the point at which some elements are highly damaged and no longer contribute to the overall stiffness of the structure. Both ductile and shear damage initiation criteria predict the initiation of damage, assuming that the initial equivalent plastic strain (${\bar{\varepsilon }}_{Damage}^{pl}$) is related to two different variables, plastic strain rate (${\dot{\bar{\varepsilon }}}^{pl}$) and stress triaxiality (η). In the case of ductile damage initiation, ductile stress triaxiality (i.e., $\eta_{D}$) is the ratio between the surface normal stress (*p*) and von Mises equivalent stress (*q*) at the start of damage as shown in Equation (3).

(3) ηD=−pq

Shear damage initiation considers the shear stress triaxiality term (i.e., $\eta_{s}$), where $k_{s}$ = 0.3 (Hooputra et al., 2004) is a dimensionless shear failure material parameter that is determined experimentally, and ($\tau_{max}$) is the maximum shear stress, Equation (4).

(4) ηs=q+kspτmax

Both damage types employ a state variable $\left(\omega \right)$ that initiates the damage when it reaches unity. The relationship is displayed Equation (5) and ω is directly proportional to the plastic deformation of the target body.

(5) $$\omega =\int \frac{d{\bar{\varepsilon }}^{pl}}{{\bar{\varepsilon }}_{Damage}^{pl}\left(\eta_{,\;}{\dot{\bar{\varepsilon }}}^{pl}\right)}=1$$

## 3. Results

### 3.1. Crab Species and Habitat Descriptions

**Figure 6:**
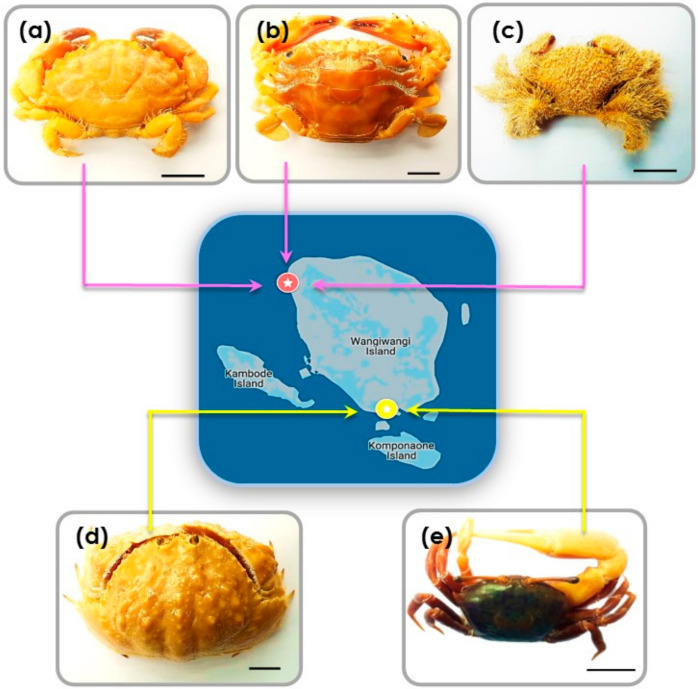
Representative examples of crabs collected from both Sombu beach and Liya beach: (**a**) *L. sanguineus*; (**b**) *Thalamita* sp., here: *T. danae*; (**c**) *P. vespertilio*; (**d**) *C. hepatica*; (**e**) *U. tetragonon*. Scale bars = 10 mm.

At Sombu beach (05°16′12.2′′ S 123°31′08.8′′ E) *L. sanguineus* (*n* = 6) was collected from the shallow tide pools, under and inside the coral rock crevices, an area covered by seaweed. *Thalamita* species (*n* = 4) (*T. danae* and *T. prymna* (spiny rock crabs)) were collected from the mangrove swamps. *P. vespertilio* crabs (*n* = 3) were found along the rocky shores among coral rubble. At Liya beach (05°23′12.1′′ S 123°35′38.4′′ E) *C. hepatica* (*n* = 3) were found in the wetland areas near shallow water, usually hiding in the sand. *U. tetragonon* (*n* = 6) were found in the intertidal zone in the dense muddy substrate regions of the coastline. Figure 6a–e provides representative images of the crabs. Table 2 provides additional morphological details for each species.

### 3.2. Morphometric Analysis of Crab Carapaces

**Figure 7:**
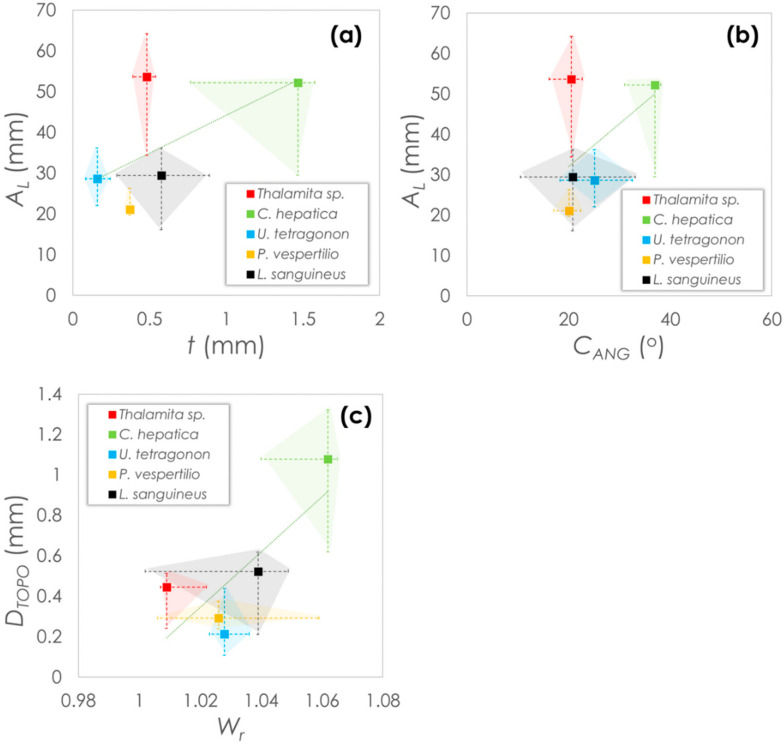
Morphological comparisons between crab species: (**a**) carapace arc length and carapace thickness, (**b**) carapace arc length and carapace angle; (**c**) topographical depth and 2D Wenzel roughness.

Morphological parameters are plotted in Figure 7 as median values with error bars indicating the maximum and minimum values within each data set. Parameters are plotted based on either the global carapace parameters (Figure 7a,b) or carapace surface parameters (Figure 7c). From the plots, it is noticeable that *Thalamita* sp. and *C. hepatica* have similarly high carapace arc lengths. However, of the two, *C. hepatica* has a higher thickness, which is up to three-fold greater than in *Thalamita* sp. and are in a similar range with *P. vespertilio* and *L. sanguineus*. *U. tetragonon* has the lowest carapace thickness, while *P. vespertilio* shows the lowest median arc length. Interestingly though, while all species show similar carapace angles (20–25° median), the carapace angle of *C. hepatica* is almost twice as high, indicating the carapace of this species is considerably less ‘flat’ than in the other species. *C. hepatica* also has the greatest topographical depth (two-fold > *L. sanguineus* (the next highest) based on median value comparisons), and the highest 2D Wenzel roughness. Mild positive correlation between the coupled variables is expected, since crab morphologies are often widely varying [33,34,35,36].

### 3.3. Cyclic Drop-Weight Impact Testing

**Figure 8:**
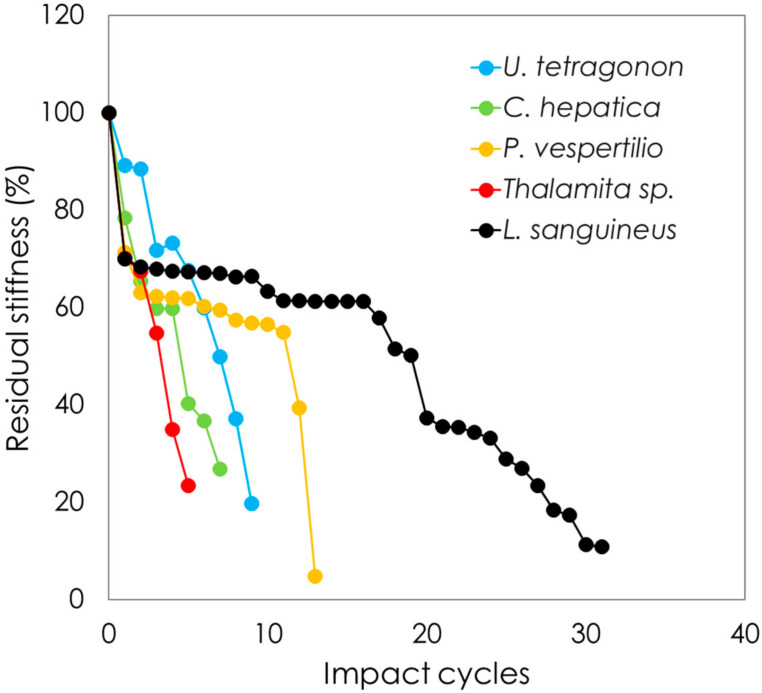
Carapace residual stiffness plotted against the number of impact cycles for each crab species (Note: *Thalamita* sp. grouped by genus; *C. hepatica* tested at 15.45 mJ after 500 impacts at 1.7 mJ and no loss in stiffness; all other species tested at 1.7 mJ).

**Figure 9:**
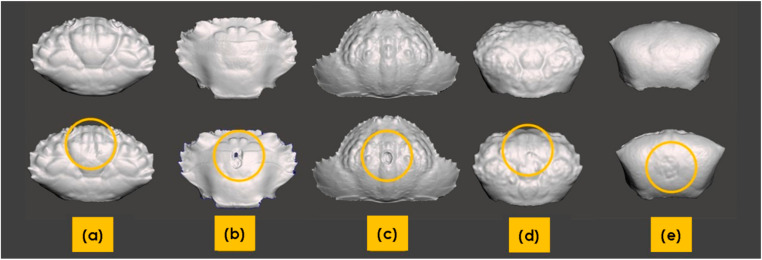
3D scan images of crab carapaces (dorsal view) before (**top**) and after (**bottom**) impact. The regions of damage are encircled in the bottom images: (**a**) *L. sanguineu*; (**b**) *Thalamita* sp. (*T. danae*); (**c**) *C. hepatica*; (**d**) *P. vespertilio*; (**e**) *U. tetragonon*.

The effects of repetitive drop weight impact testing on stiffness are plotted in Figure 8 as residual stiffness against the number of impact cycles. Each crab carapace was tested, but only representative curves are shown in this figure for visual clarity. All species were tested at 1.7 mJ, while *C. hepatica* did not show any loss in stiffness after 500 impacts at 1.7 mJ and the results shown are thus based on tests conducted at 15.45 mJ. As can be observed, all crab species lose stiffness as a function of an increasing number of impact events. While *U. tetragonon*, *C. hepatica* and *Thalamita* sp. show an almost linear decline in stiffness with impact events, *L. sanguineus* and *P. vespertilio* show sharp initial drops in stiffness under early impacts, after which the degradation rate of stiffness is seen to decrease. Dorsal face failure profiles are shown in Figure 9, from representative carapaces from each genus. *Thalamita* sp. exhibits the most brittle shear failure mode with complete through-carapace penetration from impact loading. *C. hepatica* shows a similar, albeit lesser, brittle fracture mode, with a clear shear damage profile caused by the impact loading; however, unlike in *Thalamita* sp., the steel ball does not penetrate the entire thickness of the carapace. *U. tetragonon* shows ductility in its failure, similarly to *P. vespertilio* and *L. sanguineus*. However, the extensiveness of visible denting to the dorsal face of the carapace is higher in *U. tetragonon*, followed by *P. vespertilio*, with *L. sanguineus* exhibiting the lowest levels of ductile denting as a result of cyclic impact loading. There is an interesting correlation noticeable with regard to brittle-to-ductile failure characteristics, and the residual stiffness (post-impact) of the crab carapaces. Based on the evidence presented, it is fair to postulate that brittle cracking in carapace structures (such as in *Thalamita* sp. and in *C. hepatica*) disable the solid-state continuum of the carapace, and as such, the residual stiffness decreases abruptly. Contrarily, ductile failure (denting) of carapaces allows for the retention of the carapace solid-state continuum, thereby allowing the carapace to maintain stiffness for longer.

### 3.4. Finite Element Analyses

**Figure 10:**
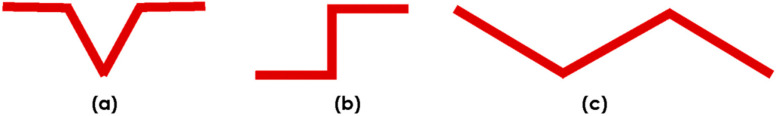
Simplified representation of carapace surface-grooves: (**a**) V-groove; (**b**) step; (**c**) zigzag.

Finite element analyses were conducted on each of the crab carapaces to complement the experimental impact testing and to further ascertain the effects of geometry on impact resistance. The models were initially validated against the experimental impact tests and were shown to be within an 8% error band for stiffness, and in defining the instance of failure. Each of the crabs has a distinctive pattern of morphological features that help to identify the species. However, there is insufficient knowledge of how the surface morphology affects the mechanical impact performance of the carapace. To address this gap in the literature, we examined the shapes of surface-grooves forming essentially, the ridges and the valleys of the carapace. The three most distinctive profiles found in the five marine crab species were (i) V-groove, (ii) step, and (iii) zigzag (cf. Figure 10).

**Figure 11:**
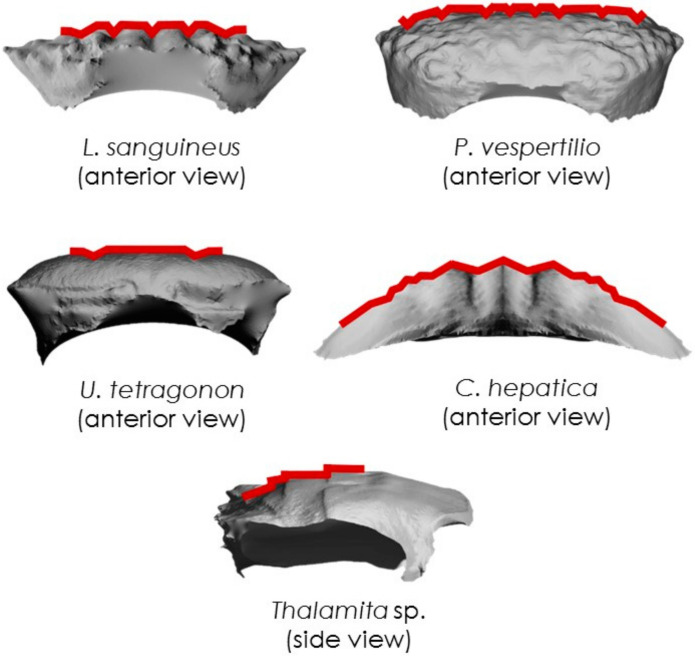
Representative examples of carapace grooves for each species.

A set of representative examples for each species is shown in Figure 11. The V-groove is a typical preponderant feature on *L. sanguineus, P. vespertilio* and *U. tetragonon* carapaces. The zigzag feature is found in *C. hepatica*, while the step is found in *Thalamita* sp. (*T. danae*). Though these features are reoccurring across the carapace, they differ in size and length. In all crab species, the most pronounced features were observed at the centre, with smaller-scale features predominating the edges of the carapace. The sizes of all three surface-grooves decrease, therefore, as a function of distance from the impact point.

**Figure 12:**
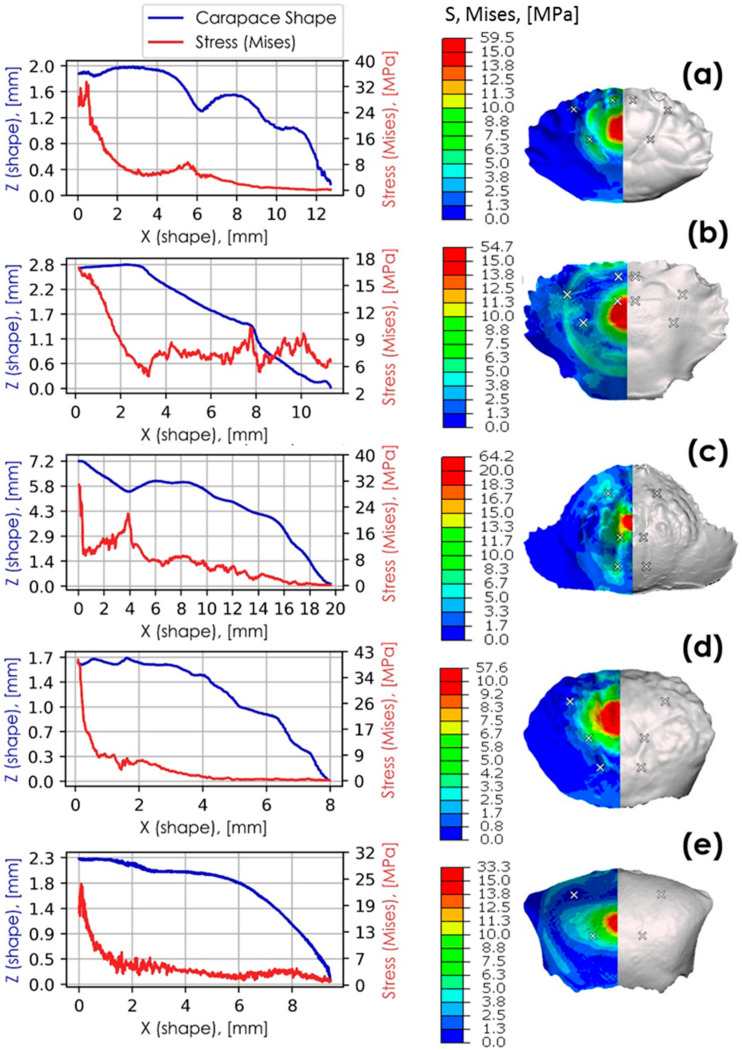
Stress (von Mises) plotted against carapace surface profiles where Z coordinates are shown in blue and stress in red. These are shown alongside surface stress profiles (von Mises) for: (**a**) *L. sanguineus* (anterior view); (**b**) Thalamita sp. (side view); (**c**) *C*. *hepatica* (anterior view); (**d**) *P*. *vespertilio* (anterior view); (**e**) *U. tetragonon* (anterior view). Carapace groove minima are identified by white crosses.

Figure 12 correlates the position of carapace grooves with distributed and localised stress. The simulation results are taken at the instance prior to failure, where half of the kinetic energy (i.e., 7.73 mJ) exerted by the impact body is transferred to the carapace. Surface von Mises stress profiles are also provided for each of the species researched. In this figure, stress distributions across the carapace surface (z-direction) peak at locations of carapace groove minima. The highest stress values are localised at the most pronounced morphological features closest to the point of impact.

The sharpest stress peaks correspond with deep zigzag grooves in *C. hepatica* at 4 mm from the centre point of impact, and with steps in *T. danae* (*Thalamita* sp.) at 7.7 mm from the centre point of impact. In both *C. hepatica* and *T. danae*, the rates of change at their localised stress peaks are high, indicating a reduced ability to dissipate localised stresses to other parts of the carapace. *L. sanguineus* has V-grooves with evidently smaller relative stress peaks at 0.8 mm and 6 mm from the centre point of impact than in the zigzag grooves of *C. hepatica*. *C. hepatica* furthermore has a much higher stress rate of change than *L. sanguineus* suggesting that *C. hepatica* is less able to dissipate stress as a function of its carapace geometry than *L. sanguineus*. Though comprising less pronounced carapace grooves, a similar trend, albeit with smaller relative stress values, can be observed in *P. vespertilio* at 1.5 mm, 3.8 mm and 5 mm from the centre point of impact. Stress peaks associated with V-grooves lessen in relative magnitude as the groove sizes decrease. Stress peaks in smaller-scale V-grooves are hardly noticeable, even if in close proximity to the point of impact (cf. *U. tetragonon* and *P. vespertilio*), suggesting that high relative stress localisations are size-dependent phenomena. Since both *C. hepatica* and *T. danae* have carapace surface morphologies that detrimentally affect the ease of stress dissipation, we suggest these aspects of their morphologies may contribute to early impact failure. Contrarily, since the other species *P. vespertilio*, *U. tetragonon* and *L. sanguineus* are relatively, more able to dissipate stress (evidenced by either the lack of stress localisation peaks, or a lower rate of change from a stress localisation peak), we suggest that these morphological characteristics of their carapaces may prolong impact failure.

Similar stress distributions can be observed in corrugated geometries that have high impact resistance [37,38]. Geometries with sharp gradients (bends) deform primarily at the bends when loaded. This observation aligns with the cyclic impact test results of both *C. hepatica* and *T. danae*, where sharp stress peaks reduced the ability of these carapaces to dissipate localised stresses and may be a primary morphological reason for observed brittle modes of failure in these species. These physical characteristics are analogous to observations made of the crab carapace simulations, but a primary difference is that whereas corrugated impact softening geometries are typically made from elastic material, crab shells are mineralised, and are inherently brittle [5,11,39]. Carapace grooves act essentially as hinges as they elastically deform in a geometrically predefined direction, which results in the localisation of stress at hinges. When these stresses reach an elastic limit, the hinges of the grooves fracture due to the brittle nature of the carapaces. The grooves of *C. hepatica* and *T. danae* carapaces are either prominent steps or zigzags, and these topographies give rise to brittle failure, in contrast to regular V-grooves observed in two of the other species, which encourage ductile (denting) failure. The sizes and arrangements of carapace grooves conceivably control the absorption and dissipation of impact energies. Small numbers of pronounced grooves lead to brittle failure modes, while shallow grooves distributed about the surface result in prolonged failure and improved overall carapace toughness. It could additionally be argued that micro-cracks build up and connect quickly as a result of stress-confinement in grooves (such as in *C. hepatica* and *T. danae* carapaces), resulting in brittle failure.

As mentioned previously, corrugated elastic materials are already understood to dampen impact. In this paper, we research brittle carapace structures and find that in such materials, corrugations, or deep grooves, result in high stress concentrations leading to early brittle failure. This is an important design consideration in areas where brittle materials are subjected to impact stresses. Dental composites are good examples as these are typically moulded to mimic the surface shape of a tooth, and are subjected to impact forces during mastication. Examples of corrugated surfaces can also be found in aerospace technologies e.g., skins, which can be manufactured from fibre-reinforced plastics. These may in turn be subjected to cyclic forces (causing fatigue), in general, and may also be damaged by impact events such as bird-strike. Impact events can also affect corrugated roof tiling, thin film particulate coatings on irregular surfaces, the surface contours of military helmets, ceramic-based prosthetics, and others. In each of the above-mentioned cases, our research findings suggest that shape is a key design parameter, where numerous and smaller corrugations/grooves on a surface are expected to improve the life-time use of a brittle material under impact more effectively than deeper corrugations/grooves on a surface.

## 4. Conclusions

The shapes of crab carapaces influence their failure modes under impact. In this work, carapaces from five genera were tested and modelled to ascertain whether carapace morphology could influence carapace behaviour under impact loading. Cyclic impact resistance in order of highest to lowest can be listed as follows in the species tested: *L. sanguineus* > *P. vespertilio* > *U. tetragonon* > *C. hepatica* > *Thalamita* sp., with *L. sanguineus* and *P. vespertilio* showing ductile characteristics, *U. tetragonon* showing a mixed ductile–brittle failure mode, and *C. hepatica* and *Thalamita* sp. failing in a highly brittle manner. Crab species with brittle failure characteristics exhibit both the greatest arc lengths and the deepest V-grooves. Crab species with ductile (denting) failure modes have shorter arc lengths and smaller more broadly distributed carapace grooves. Deep carapace grooves heighten the localisation of stresses and enable extended deformations, but concurrently deep grooves decrease stress dissipation through the carapace, which may lead to premature brittle failure under cyclic impact. Decreased depths of carapace grooves distributed more broadly across the surface, alleviates high localised stress concentrations and enables stress dissipation through the entire body of the carapace. The findings of this paper provide new insights into the geometrical design of impact resistance in rigid biomimetic structures, including but not limited to: composite dental moulds, corrugated aerospace skins, and contoured military armour.

## Figures and Tables

**Table 1:** Mesh specifications for each model.

Model	No of Elements	Element Type	Fine Edge Mesh Size (mm)	Coarse Edge Mesh Size (mm)
*L. sanguineus*	60,618	S3R	0.12	0.27
*P. vespertilio*	53,849	S3R	0.08	0.2
*U. tetragonon*	55,132	S3R	0.07	0.2
*C. hepatica*	75,490	S3R	0.12	0.63
*T. danae*	59,741	S3R	0.12	0.27

**Table 2:** Additional morphological details for each species.

Species	Morphologies
*L. sanguineus*	The carapace is wider than it is long and is anteriorly convex. Markings no the dorsal face of the carapace surface are unclear. The antero-lateral margins have six teeth. The chelae fingers are spoon-like in shape, and the chelae fingers are black in colour.
*T. danae* & *T. prymna*	The carapace is transversely ovate with five antero-lateral teeth. The fronto-orbital margin is very wide. *T. danae* and *T. prymna* are of a very similar morphology. The main distinction is the basal antennal segment joints of *T. prymna*, which are without spines, unlike in *T. danae*.
*P. vespertilio*	The carapace is marginally little longer than it is wide. The antero-lateral margin of the carapace has three teeth. The carapace is covered in, as are the chelae and legs. The lower half of the chela (outside face) is covered by bead-like granules.
*C. hepatica*	Posterolateral parts of the carapace with clypeiform expansion covers the crab’s legs. Its margin has teeth, laterally the margin is dentate. The anterior edge of this clypeiform structure is gently denticulate. The right (larger) chela exhibits a specialised cutting tooth.
*U. tetragonon*	The frontal region of the carapace is narrower at its base than at its tip. Sexual dimorphism between male and female chelae is obvious since the outer face of male (larger) chela is granulated.
